# Vascular complications in adult postcardiotomy cardiogenic shock patients receiving venoarterial extracorporeal membrane oxygenation

**DOI:** 10.1186/s13613-018-0417-3

**Published:** 2018-06-19

**Authors:** Feng Yang, Dengbang Hou, Jinhong Wang, Yongchao Cui, Xiaomeng Wang, Zhichen Xing, Chunjing Jiang, Xing Hao, Zhongtao Du, Xiaofang Yang, Yu Jiang, Xiaotong Hou

**Affiliations:** Center for Cardiac Intensive Care, Beijing Institute of Heart, Lung, and Blood Vessels Diseases, Beijing Anzhen Hospital, Capital Medical University, No. 2 Anzhen Rd, Chaoyang District, Beijing, 100029 China

**Keywords:** Postcardiotomy cardiogenic shock, Venoarterial extracorporeal membrane oxygenation, Complications, Cannulation, Survival

## Abstract

**Background:**

The rate, prognostic impacts, and predisposing factors of major vascular complications (MVCs) in patients underwent venoarterial extracorporeal membrane oxygenation (VA-ECMO) by surgical cut-down are poorly understood. The purpose of this study was to identify these parameters in adult VA-ECMO patients.

**Methods:**

Adult postcardiotomy cardiogenic shock (PCS) patients receiving VA-ECMO by femoral surgical cut-down cannulation from January 2004 to December 2015 were enrolled in this study. Patients were separated into two groups depending on the presence of MVCs. Multivariate logistic regression was performed to identify factors independently associated with MVCs.

**Results:**

Of 432 patients with PCS treated with VA-ECMO, 252 patients (58.3%) were weaned off VA-ECMO and 153 patients (35.4%) survived to discharge. MVCs were seen in 72 patients (16.7%), including bleeding or hematoma in the cannulation site (8.6%), limb ischemia requiring fasciotomy (8.6%), femoral artery embolism (0.7%), and retroperitoneal bleeding (0.7%). The rate of survival to discharge was 16.7 and 39.2% in patients with or without MVCs, respectively (*p *< 0.001). Obesity, concomitant with intra-aortic balloon pump (IABP), Sequential Organ Failure Assessment (SOFA) score at 24 h post-ECMO, and hemostasis disorder were shown to be associated with MVCs. MVCs were an independent risk factor for in-hospital mortality by multivariate analysis (odds ratio 3.91; 95% confidence interval, 1.67–9.14; *p *= 0.013).

**Conclusions:**

MVCs are common and associated with higher in-hospital mortality among adult PCS patients receiving peripheral VA-ECMO support. The obesity, concomitant with IABP, SOFA score at 24 h post-ECMO, and hemostasis disorder were independent risk factor of MVCs.

## Background

Postcardiotomy cardiogenic shock (PCS) remains a clinical challenge, with high mortality rate [1]. Venoarterial extracorporeal membrane oxygenation (VA-ECMO) may provide a survival benefit for patients with PCS. Femoral VA-ECMO is less invasive and rapidly instituted at the bedside, especially in patients who have had a cardiac arrest [2]. The use of VA-ECMO for adult PCS has increased, with a survival rate of 16–42% [3–7].

Successful cannulation is the prerequisite and basis for VA-ECMO support for achieving good clinical results. Percutaneous and surgical cut-down vascular cannulations are commonly performed for VA-ECMO implantation [8, 9]. Major vascular complications (MVCs) can occur from cannulation of the femoral vessels. However, the actual prevalence of MVCs and outcomes of PCS patients underwent VA-ECMO by surgical cut-down is still unclear. To date, only a few studies have reported on MVCs in PCS patients from single-center experience [10–12]. Therefore, we elucidated the prevalence of MVCs and their impact on in-hospital mortality in adult PCS patients receiving peripheral VA-ECMO by surgical cut-down. Furthermore, we also assessed the possible risk factors associated with the occurrence of MVCs.

## Methods

### Study population

Between January 2004 and December 2015, 43,192 adult patients underwent cardiac surgery. Of these patients, 451 patients (1.0%) required VA-ECMO support due to failure to wean from cardiopulmonary bypass (CPB) (*n* = 231) or a refractory PCS in the intensive care unit (ICU) (*n* = 220). For all patients receiving VA-ECMO, preoperative, perioperative, and postoperative clinical variables were prospectively recorded in the institutional database.

**Fig. 1:**
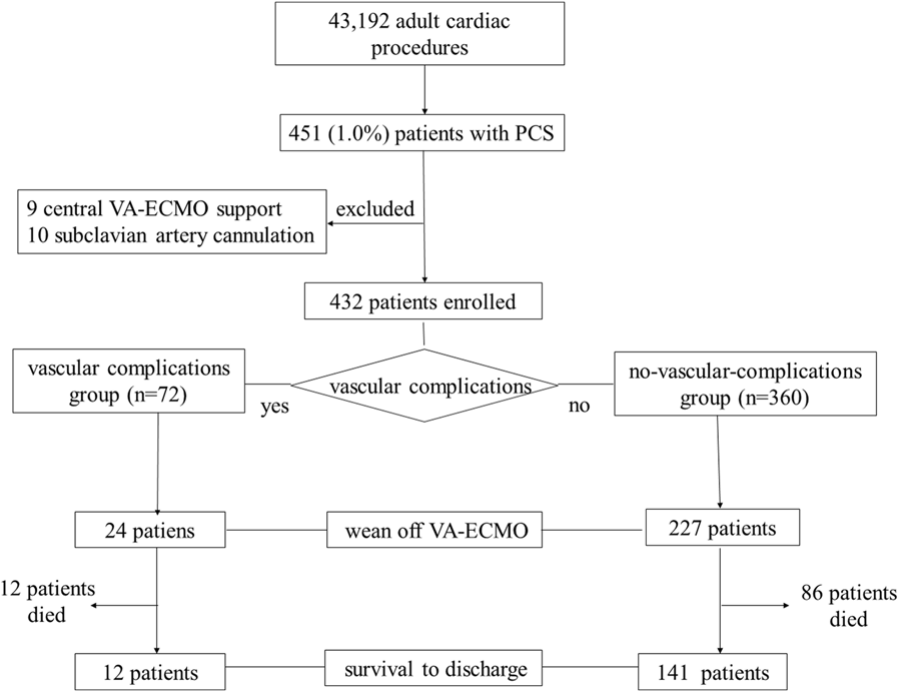
Study flowchart. A total of 43,192 adult patients undergoing cardiac surgery were screened. Of these patients, 432 patients who received VA-ECMO by means of femoral surgical cut-down cannulation for cardiac support were enrolled. Patients were divided into those who had or who had not had major vascular complications ([vascular complications group, *n* = 72] and [control group, *n* = 360]). Rates of in-hospital mortality, wean off VA-ECMO, and morbidity were compared

Patients who received VA-ECMO by femoral surgical cut-down cannulation for cardiac support (*n* = 432) were enrolled in this study. The patients implanted with VA-ECMO by either central (*n* = 9) or subclavian (*n* = 10) artery cannulation approach were excluded (Fig. 1). Patients were divided into two groups (MVCs group [*n* = 72] and no-MVCs group [*n* = 360]). The study was approved by the institutional ethics committee/review board of the Beijing Anzhen Hospital, and the requirement for informed patient consent was waived in view of the retrospective nature of the study.

### ECMO implantation techniques

VA-ECMO cannulas were surgically inserted by trained ECMO team members with femoral–femoral approach. The groin was incised, and the common femoral artery and vein were identified. A needle was inserted into the femoral vein using a subcutaneous tunnel. Following the Seldinger technique, a guidewire was advanced from the femoral vein toward the right atrium. The femoral vein was progressively dilated. A Bio-Medicus 19Fr-21Fr cannula was introduced over the guidewire, with placement of the tip just proximal to the right atrium. The common femoral artery was then similarly cannulated with Bio-Medicus 15Fr or 17Fr cannula. An additional 6F catheter was also performed at the time of ECMO initiation to preserve limb perfusion in most of patients (96.3%). All patients were cannulated and implanted the distal perfusion catheter under echocardiography guidance. In 246 patients (56.9%), a 7.5F IABP catheter (Datascope Corp., Fairfield, NJ, USA) was placed percutaneously through the contralateral femoral artery.

### Patient management

Detailed management strategies for patients were previously described [13]. ECMO blood flow was adjusted to maintain mixed venous oxygen saturation (SvO_2_) level of 75%. The bleeding at the femoral cannulation site and the blood circulation of the lower limbs were observed continuously with trained ICU staff (bedside nurse) during ECMO support.

Heparin is the most commonly used anticoagulant. Previously given heparin was reversed with protamine prior to initiating VA-ECMO when patients had failed to wean from CPB. A heparin bolus (5000 IU) was injected before cannulation for PCS patients in the ICU. After VA-ECMO support, if surgical bleeding could be controlled, the patients were given continuous intravenous infusion of unfractionated heparin as early as possible to maintain an activated clotting time (ACT) of 160–180 s. Packed red blood cells were administered if the hemoglobin levels were less than 8 g/dL. Platelets were administered to maintain the platelet count at more than 50,000 × 10^9^/L. When the patient had clinically improved, a weaning trial was performed using the protocol previously described [13]. All cannula removals were performed after exposing the femoral vessels. The femoral artery and vein were primarily repaired.

### Main aims and definitions

The primary endpoint was in-hospital mortality. The secondary endpoints were the proportion of patients weaned from VA-ECMO and major postoperative complications. In-hospital mortality was defined as death from any cause occurring in the hospital after surgery. Weaning off ECMO was considered successful when a patient survived VA-ECMO explantation for longer than 48 h [14].

MVCs related to cannulation were defined as those required surgical intervention by previous studies [7, 10, 11, 15]. Surgical indications included bleeding or hematoma at the VA-ECMO cannulation site, severe limb ischemia, femoral artery embolism, and retroperitoneal bleeding. Severe limb ischemia complications were defined as the deterioration of lower limb circulation ipsilateral to the cannulation site requiring surgical intervention (thrombectomy, fasciotomy, or amputation).

Postoperative renal failure was diagnosed in the presence of oliguria (< 30 mL/h) and a doubling of postoperative creatinine values requiring continuous renal replacement treatment (CRRT). Hemostasis disorders during ECMO included platelets < 20 × 10^9^/L, fibrinogen < 1.5 g/L, and prothrombin time < 30% of the standard value [16]. Neurologic complications were recorded in the presence of clinical or radiologic evidence for a new neurologic deficit that was not present preoperatively. Diagnosis of acute extremity compartment syndrome is based on clinical symptoms and/or intra-compartmental pressure [17].

### Statistical analysis

Categorical variables and frequencies were presented as percentages and continuous variables as mean (range) or median (interquartile range) according to their distribution. Normality of distribution was tested with Kolmogorov–Smirnov test. The variables for patients with and without MVCs were compared using Student’s *t* test or the Mann–Whitney *U* test for continuous variables, and Chi-square or Fisher’s exact tests for categorical variables. The logistic regression analysis identified predictors of MVCs through the enter method. Survival rates were calculated using the Kaplan–Meier method. Analyses were performed using SPSS 20.0 (SPSS, Inc., Chicago, IL, USA) software and a two-sided *p *< 0.05 defined significance.

## Results

### Patient demographics, pre-ECMO characteristics, and ECMO variables

Demographics and pre-ECMO risk profiles of the 432 VA-ECMO patients are illustrated in Table 1. ECMO patients with MVCs had bigger body surface area (BSA), higher body mass index (BMI), and higher inotrope scores at the beginning of VA-ECMO (*p *< 0.05). It is worth noting that the incidence of MVCs in patients with congenital heart disease was lower, but the difference was not statistically significant (*p *= 0.068).

**Table 1:** Patient demographics, comorbidities, and surgical procedures

Variable	With vascular complications (*n* = 72) *n* (%)	Without vascular complications (*n* = 360) *n* (%)	*P* value
*Baseline characteristics*			
Male	52 (72.2%)	233 (64.7%)	0.220
Age (years)	57(48.3, 65.0)	57(47.3, 65.0)	0.905
Older age (≥ 65 years)	19 (26.4%)	101 (28.1%)	0.773
BSA (m^2^)	1.9 (1.8, 2.0)	1.8 (1.7, 2.0)	0.009
BMI (kg/m^2^)	25.0 ± 3.7	23.6 ± 3.4	0.002
Smoking	34 (47.2%)	145 (40.3%)	0.275
*Comorbidities*			
Coronary artery disease	42 (58.3%)	176 (48.9%)	0.143
Peripheral vascular disease	12 (16.7%)	46 (12.8%)	0.377
Hypertension	32 (44.4%)	140 (38.9%)	0.356
Diabetes	15 (20.8%)	75 (20.8%)	1.000
Hypercholesterolemia	61 (8.5%)	260 (7.2%)	0.718
Chronic obstructive lung disease	10 (1.4%)	50 (1.4%)	0.998
Liver dysfunction	10 (1.4%)	2 (0.6%)	0.437
APACHE II score	32.8 ± 5.2	30.8 ± 7.1	0.448
*Type of surgery*			
CABG	35 (48.6%)	142 (39.4%)	0.149
Valve procedure	17 (23.6%)	105 (29.2%)	0.339
CABG + valve procedure	8 (11.1%)	37 (10.3%)	0.833
Congenital heart disease	0	16 (4.4%)	0.068
Repair of acute aortic dissection	4 (5.6%)	18 (5.0%)	0.845
Repair of acute aortic dissection + CABG	4 (5.6%)	8 (2.2%)	0.116
Heart transplantation	3 (4.2%)	18 (5.0%)	0.761
Pulmonary embolectomy	1 (1.4%)	9 (2.5%)	0.567
Others	0	6 (1.7%)	0.270
Reoperation	20 (2.8%)	12 (3.3%)	0.808

*APACHE* acute physiologic and chronic health evaluation, *BSA* body surface area, *BMI* body mass index, *CABG* coronary artery bypass grafting, *CHD* congenital heart disease, *CPB* cardiopulmonary bypass, *CPR* cardiopulmonary resuscitation

Compared with the control group, blood lactate, and sequential organ failure assessment (SOFA) score at ECMO initiation, the ratio of severe bleeding (22.2 vs. 13.1%, *p *= 0.044) and repeat thoracotomy (51.4 vs. 36.7%, *p *= 0.019) in patients with MVCs were statistically higher. Therefore, patients with MVCs required a significantly higher number of red blood cell transfusion (*p *= 0.002). The duration of mechanical-assisted ventilation, ICU stay, and the length of stay in-hospital for MVCs patients were significantly shorter (Table 2).

**Table 2:** ECMO details and outcomes

Variable	With vascular complications (*n* = 72) *n* (%)	Without vascular complications (*n* = 360) *n* (%)	*P* value
*ECMO implantation*			
Failure to wean off CPB	40 (55.6%)	198 (55.0%)	0.932
LCOS in ICU	32 (44.4%)	162 (45.0%)	0.932
Inotrope scores	52.5 ± 11.2	38.3 ± 12.9	0.008
Lactate at ECMO initiation (mmol/L)	11.0 (7.8, 14.3)	9.5 (6.9, 12.8)	0.035
Peak lactate during ECMO (mmol/L)	16.2 (13.6, 20.8)	15.2 (11.5, 18.5)	0.004
SOFA score at ECMO initiation	13.0 (12.0, 13.0)	12.0 (11.0, 13.0)	< 0.001
SOFA score at 24 h post-ECMO	11.0 (10.0, 11.0)	9.0 (7.0, 11.0)	< 0.001
Arterial cannula size, Fr	16.5 ± 0.6	16.1 ± 0.7	0.467
Ongoing CPR	13 (18.1%)	56 (15.6%)	0.597
IABP support	45 (62.5%)	200 (55.6%)	0.280
*ECMO outcomes*			
Weaning from ECMO	24 (33.3%)	228 (63.3%)	< 0.001
Survival to discharge	12 (16.7%)	141 (39.2%)	< 0.001
Duration of ECMO (days)	2.9 (1.6, 5.4)	3.8 (2.2, 5.5)	0.204
*Complications*			
Renal failure required CRRT	38 (52.1%)	171 (47.6%)	0.490
Neurologic complications	11 (15.3%)	56 (15.6%)	0.953
DIC	3 (4.2%)	6 (1.7%)	0.177
Severe bleeding	16 (22.2%)	47 (13.1%)	0.044
Tracheostomy	27 (37.5%)	140 (39.0%)	0.812
Repeat thoracotomy	37 (51.4%)	132 (36.7%)	0.019
Femoral site infection	6 (8.3%)	25 (7.0%)	0.681
Sepsis	13 (18.1%)	84 (23.3%)	0.335
*Medical resources*			
PRBC transfusion (units)	28.0 (19.3, 35.8)	22.0 (14.0, 32.0)	0.002
FFP	2400 (1600, 3600)	2000 (1400, 3000)	0.076
Platelets	3.0 (1.0, 5.0)	3.0 (1.0, 5.0)	0.578
Duration of MV	94.5 (38.8, 191.3)	120.0 (51.0, 210.0)	0.018
ICU stay (days)	107.0 (44.8, 237.4)	168.0 (95.0, 255.8)	0.007
Post-ECMO hospital stay (days)	0 (0, 7.8)	7 (0, 15.8)	< 0.001
Hospital stay (days)	17.0 (11.0, 25.8)	23.0 (16.0, 34.8)	0.001

Data presented as *n* (%) categorical variables and median (interquartile range) for non-parametric variable. Inotrope scores = dosage of dopamine (in μg/kg/min) + dosages of dobutamine (in μg/kg/min) + [dosages of epinephrine (in μg/kg/min + norepinephrine (in μg/kg/min)] × 100 + dosages of pituitrin (in u/min) × 100 + dosages of milrinone (in μg/kg/min) × 15

*CPR* cardiopulmonary resuscitation, *CRRT* continuous renal replacement therapy, *DIC* disseminated intravascular coagulation, *ECMO* extracorporeal membrane oxygenation, *FFP* fresh frozen plasma, *ICU* intensive care unit, *LCOS* low cardiac output syndrome, *MV* mechanical ventilation, *PRBC* packed red blood cells, *SOFA* sequential organ failure assessment

### Occurrence rate of MVCs

A total of 72 patients (16.7%) had at least one episode of MVCs, including 37 patients (8.6%) with severe limb ischemia who progressed to compartment syndrome requiring prophylactic fasciotomy, 12 patients (2.8%) required limb amputation, 37 patients (8.6%) with significant bleeding or hematoma at the cannulation site that required surgical exploration, 3 patients (0.7%) with femoral artery embolism requirement surgical intervention, and 3 patients (0.7%) with retroperitoneal bleeding. Ten patients (2.3%) had both severe limb ischemia and bleeding in the cannulation site.

### Predisposing factors for MVCs

Table 3 reports the factors associated with the MVCs. The obesity, coronary artery disease, lactate and SOFA score at ECMO initiation, peak lactate during ECMO, SOFA score at 24 h post-ECMO, and concomitant with IABP were the risk factors significantly associated with the severe limb ischemia in the univariable analysis, whereas the multivariable logistic regression analysis retained obesity, SOFA score at 24 h post-ECMO, and VA-ECMO combined with IABP as the risk factors independently associated with the severe limb ischemia. In addition, the hemostasis disorders were significantly associated with cannulation site bleeding/hematoma during VA-ECMO support.

**Table 3:** Univariable and multivariable analyses of factors associated with major vascular complications (severe limb ischemia and cannulation site bleeding)

Factor	Univariable analysis OR [95% CI], *p* value	Multivariable analysis OR [95% CI], *p* value
*Severe limb ischemia*		
Peripheral artery disease	0.73 [0.37–1.46] 0.378	
Hypertension	0.79 [0.47–1.31] 0.356	
Hypercholesterolemia	0.84 [0.33–2.13] 0.718	
Smoking	0.75 [0.45–1.25] 0.276	
Obesity	3.47 [1.20–10.03] 0.005	2.65 [1.26–5.56] 0.010
Coronary artery disease	2.47 [1.19–5.14] 0.022	
Diabetes	0.86 [0.68–3.14] 0.326	
Combined with IABP	2.97 [1.33–6.67] 0.018	2.49 [1.19–2.65] 0.025
ECPR	0.83 [0.43–1.63] 0.597	
Lactate at ECMO initiation	1.07 [0.98–1.14] 0.052	
Peak lactate during ECMO	1.10 [1.03–1.16] 0.002	
SOFA score at ECMO initiation	2.09 [1.60–2.23] < 0.001	
SOFA score at 24 h post-ECMO	1.67 [1.41–1.98] < 0.001	1.43 [1.08–1.86] 0.010
*Cannulation site bleeding*		
Hemostasis disorders during ECMO	7.21 [2.28–22.77] < 0.001	6.11 [1.88–19.87] < 0.001

*CNS* central nervous system complications, *CRRT* continuous renal replacement treatment, *DIC* disseminated intravascular coagulation, *ECMO* extracorporeal membrane oxygenation, *ECPR* extracorporeal cardiopulmonary resuscitation, *IABP* intra-aortic balloon pump, *SOFA* sequential organ failure assessment

### Impact of MVCs on survival

**Fig. 2:**
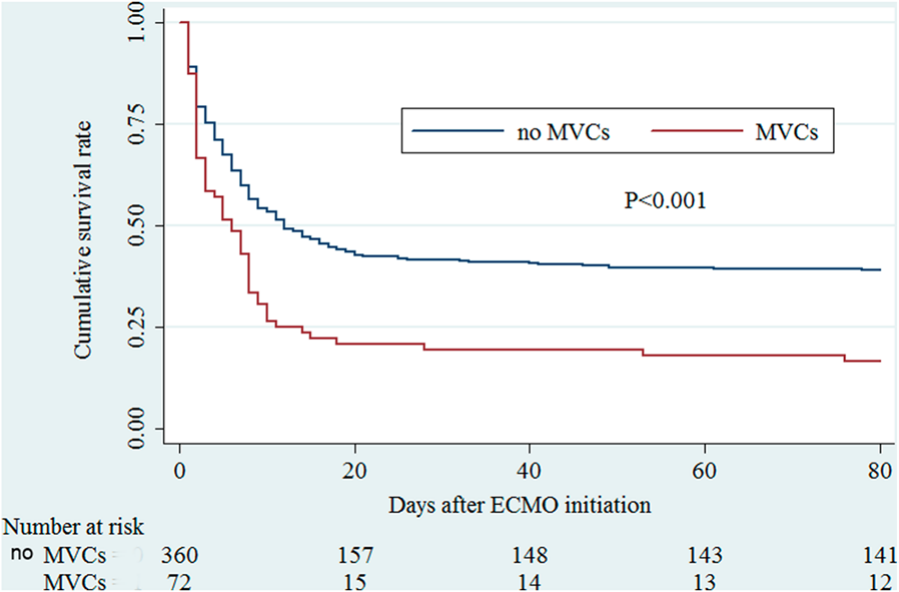
Kaplan–Meier cumulative in-hospital mortality after ECMO support. Kaplan–Meier survival curves show in-hospital mortality in patients with major vascular complications (red lines) and without major vascular complications (black lines) (*p *< 0.001)

The MVCs had a significant impact on in-hospital mortality (Fig. 2). Survival was 16.7% for patients with MVCs, compared with 39.2% for patients without MVCs (*p *< 0.001). The rates of weaning off VA-ECMO for the patients with MVCs were also lower than for those patients without MVCs (33.3 vs. 63.3%, *p *< 0.001).

Table 4 shows predisposing factors that influenced in-hospital mortality significantly by multivariate analysis. Presence of MVCs, renal dysfunction requiring CRRT, severe bleeding, and neurologic complications were independent risk factors associated with in-hospital mortality.

**Table 4:** Predisposing factors for in-hospital mortality (multivariate logistic regression)

Variable	Odds ratio	95% confidence interval	*p* value
Major vascular complications	3.91	1.67–9.14	0.013
Renal failure required CRRT	10.98	6.21–19.41	< 0.001
Severe bleeding	15.86	3.61–69.63	< 0.001
Neurologic complications	13.68	5.38–34.80	< 0.001

*CRRT* continuous renal replacement treatment

## Discussion

### Prevalence of MVCs in adult PCS patients

To our knowledge, this is the largest study on the MVCs in adult PCS patients receiving femoral–femoral VA-ECMO support by surgical cut-down. We observed that MVCs occurred in 16.7% of cases, including severe limb ischemia (8.6%) and bleeding in cannulation site (8.6%), which are in accordance with the literature on MVCs occurring in VA-ECMO patients (4.7–20.0%), largely due to lack of a clear definition [10, 11, 18–21].

Severe limb ischemia complications occurred in 8.6% of all our patients. In previous studies, others reported limb ischemia events among 2.3–52.0% of adult patients undergoing VA-ECMO [10–12, 21]. In a series of 84 adult patients, Tanaka and colleagues found that MVCs requiring surgical intervention were seen in 17 patients (20%), including 10 patients (12%) who had distal limb ischemia requiring prophylactic fasciotomy [11]. Similarly, another study in 93 adult PCS patients receiving peripheral VA-ECMO found that 15.1% of patients had severe limb ischemia [20]. In a meta-analysis of 1866 adult patients with CS, Cheng and colleagues [7] found that severe limb ischemia occurred in 10.3% of patients. Compared with these results, our observed limb ischemia rate of 8.6% is low, which may in part be explained by the potential advantages of surgically inserted with a distal perfusion catheter.

The mechanism of severe limb ischemia in PCS patients undergoing VA-ECMO is still unclear. A cannula in the common femoral artery has the potential of obstructing flow to the lower limb, and therefore reducing blood perfusion distal to the puncture site. VA-ECMO requires a larger arterial cannula in the femoral artery for delivering oxygenated blood to the patient. There are several reports on prophylactic insertion of a small anterograde perfusion cannula into the superficial femoral artery, which can reduce the incidence of severe limb ischemia [13, 21, 22]. Although the incidence of severe limb ischemia was low, 8.6% of patients still needed decompression drainage, and 2.6% of patients required amputation in this study. There may be other reasons for the occurrence of limb ischemia complication, and further research is needed.

Another one of the most common MVCs is bleeding in the cannulation site, with a rate of 18.5% in peripheral VA-ECMO according to the extracorporeal life support registry [23]. A reduction in platelet count, hemolysis, and a consumptive coagulopathy along with systemic heparinization can further increase the hemorrhagic risk during VA-ECMO support. These patients required more blood transfusion; therefore, the effects of VA-ECMO support were severely affected.

### Hospital outcome in adult VA-ECMO patients experiencing MVCs

There is still some controversy about the effect of MVCs on hospital outcomes. In this study, MVCs were an independent risk factor for in-hospital mortality in patients undergoing VA-ECMO support. This is in accordance with Tanaka [10] and colleagues, who demonstrated a strong relationship between vascular complications and in-hospital mortality. In contrast, in 143 patients receiving VA-ECMO support by femoral cannulation, of those 17 (11.9%) observed vascular complications. Two patients (1.4%) who had extremity ischemia required limb amputation. However, the MVCs were not associated with early mortality (65 vs. 61%, *p *= 0.95) [11]. The MVCs patients had increased transfusion requirements in this study as they are not only bleeding at the cannulation site, but they are also bleeding at the compartment syndrome decompression site. In previous studies, others found a strong relationship between red blood cell transfusion during VA-ECMO and in-hospital mortality [24, 25]. In agreement with other previous reports, we also found that neurologic complications, severe bleeding, ECPR, and renal failure requiring renal replacement therapy were associated with dismal prognoses [3, 4].

### Predictors of MVCs in adult VA-ECMO patients

Given the poor outcomes associated with MVCs, identifying the risk factors and actively preventing MVCs are very important for these patients. It is worth noting that obesity, concomitant IABP, and SOFA score at 24 h post-ECMO appeared to be significant risk factors for severe limb ischemia. Intriguingly, peripheral arterial disease was not associated with limb ischemia (*p *= 0.38) in this study. The peripheral arterial disease and absence of a distal perfusion catheter has been found to be predictors of severe limb ischemia in previous studies [10, 11]. All PCS patients receiving VA-ECMO support were implanted by surgical cut-down and conventional placement of a distal perfusion catheter in this study. Therefore, for the patients with femoral artery stenosis (moderate to severe), surgical cut-down combined with a distal perfusion tube for implantation of VA-ECMO is recommended. Although the incidence of limb ischemia was generally low, IABP has the risk of increasing the risk of limb ischemia [26]. A total of 246 patients (56.9%) received VA-ECMO combined with IABP support. A total of 235 of these patients (95.5%) received IABP first but were still unable to maintain hemodynamic stability, and were then given VA-ECMO. The other 11 patients (4.5%) underwent VA-ECMO first, and received IABP for that opening of the aortic valve were restricted. Therefore, it is necessary to pay attention to the occurrence of limb ischemia when the combined with IABP support is needed. It is worth noting that obesity was a significant risk factor for severe limb ischemia. Although the severe limb ischemia affects the clinical prognosis of those patients, there is still no suitable time for surgical decompression and drainage. In the present study, we found that higher SOFA score at 24 h post-ECMO was associated with increased incidence of MVCs. The clinical conditions of patients with higher SOFA score were more severe, and distal tissue hypoperfusion and thrombocytopenia seemed to be more common in those patients, which might account for our findings. In addition, we also demonstrated that the hemostasis disorders were independently associated with bleeding/hematoma in cannulation site during VA-ECMO support. Further research should focus on prevention and early management of MVCs to avoid devastating consequences.

Our study also has limitations. First, it is a retrospective, single-center study. Second, although the number of patients included in this study is small, our data report the largest series of adult PCS patients receiving VA-ECMO by surgical cut-down for evaluating the impact of MVCs on outcome and analysis of associated factors. The conclusions of this study can provide reference for peers. Third, we did not perform follow-up. Further studies focusing on this point are needed to support long-term safety of surgically inserted.

## Conclusions

The MVCs in adult PCS patients undergoing VA-ECMO support by surgical cut-down are common. Furthermore, the MVCs were associated with higher in-hospital mortality rates. The obesity, SOFA score at 24Â h post-ECMO, and the concomitant with IABP were independent risk factors for the occurrence of limb ischemia in VA-ECMO support. Surgical cut-down implantation of the VA-ECMO technique might be considered a valuable option for adult PCS patients, although more data and larger patient cohorts are needed to confirm the findings presented herein.

## Abbreviations

AMI: acute myocardial infarction
CABG: coronary artery bypass grafting
CAD: coronary artery disease
CI: confidence interval
CPB: cardiopulmonary bypass
CPR: cardiopulmonary resuscitation
ECMO: extracorporeal membrane oxygenation
ECPR: extracorporeal cardiopulmonary resuscitation
FFP: fresh frozen plasma
HR: hazard ratio
Htx: heart transplantation
IABP: intra-aortic balloon pump
LCOS: low cardiac output syndrome
LOS: length of stay
LV: left ventricular
LVEF: left ventricular ejection fraction
MVCs: major vascular complications
PLT: platelet
PRBC: packed red blood cells
VAD: ventricular assist device
SvO_2_: mixed venous oxygen saturation
SOFA: sequential organ failure assessment
VA-ECMO: venoarterial extracorporeal membrane oxygenation
